# Application of Green Technology to Extract Clean and Safe Bioactive Compounds from *Tetradesmus obliquus* Biomass Grown in Poultry Wastewater

**DOI:** 10.3390/molecules28052397

**Published:** 2023-03-06

**Authors:** Jelena Vladić, Jelena Molnar Jazić, Alice Ferreira, Snežana Maletić, Dragoljub Cvetković, Jasmina Agbaba, Senka Vidović, Luisa Gouveia

**Affiliations:** 1 Department of Biotechnology and Pharmaceutical Engineering, Faculty of Technology, University of Novi Sad, 21000 Novi Sad, Serbia; 2 Department of Chemistry, Biochemistry and Environmental Protection, Faculty of Sciences, University of Novi Sad, 21000 Novi Sad, Serbia; 3 Laboratório Nacional de Energia e Geologia IP, UBB-Bioenergy and Biorefineries Unit, Estrada Paço Lumiar, Edificio G, 1649-038 Lisbon, Portugal; 4 GreenCoLab—Green Ocean Technologies and Products Collaborative Laboratory, Centro de Ciências do Mar do Algarve, Universidade do Algarve Campus Gambelas, 8005-139 Faro, Portugal

**Keywords:** subcritical water extraction, nutrient recycling, microalgae, metal removal, antioxidants, organic profile

## Abstract

Microalgae are capable of assimilating nutrients from wastewater (WW), producing clean water and biomass rich in bioactive compounds that need to be recovered from inside the microalgal cell. This work investigated subcritical water (SW) extraction to collect high-value compounds from the microalga *Tetradesmus obliquus* after treating poultry WW. The treatment efficiency was evaluated in terms of total Kjeldahl nitrogen (TKN), phosphate, chemical oxygen demand (COD) and metals. *T. obliquus* was able to remove 77% TKN, 50% phosphate, 84% COD, and metals (48–89%) within legislation values. SW extraction was performed at 170 °C and 30 bar for 10 min. SW allowed the extraction of total phenols (1.073 mg GAE/mL extract) and total flavonoids (0.111 mg CAT/mL extract) with high antioxidant activity (IC_50_ value, 7.18 µg/mL). The microalga was shown to be a source of organic compounds of commercial value (e.g., squalene). Finally, the SW conditions allowed the removal of pathogens and metals in the extracts and residues to values in accordance with legislation, assuring their safety for feed or agriculture applications.

## 1. Introduction

Livestock activities have been intensively carried out to feed the growing world population, resulting in major environmental problems, such as soil degradation, surface and groundwater contamination [1]. Water quality and shortage are central concerns of the present century, included in the 2030 Agenda and on Sustainable Development Goals. The discharge of untreated or inadequately treated effluents into water bodies supplies the aquatic environment with a myriad of chemical compounds that can endanger aquatic organisms directly, by triggering hazardous effects, and indirectly, by changing some physicochemical features of the medium.

The poultry sector is currently the second largest contributor to global meat production [2]. Large volumes of poultry effluents are produced in agro-industrial farms and slaughterhouses worldwide, from the slaughtering of animals, and meat processing plants [3]. They are characterized by high organic (e.g., residual blood, skin, fat, manure, etc.) and nutrient loads, suspended solids and metals (e.g., Zn, Cu, Ca, Mg, Fe, and Mn) that need to be removed before they can be discharged into the environment to avoid eutrophication risks [4,5,6]. It is estimated for the poultry industry a flowrate of WW of 21 million m^3^/year, which represents 161,000 ton/year of chemical oxygen demand (COD) and 13,000 ton/year of nitrogen for treatment [7].

Conventional wastewater treatment (WWT) has allowed the removal of pollutants but at the expenses of high-energy input (e.g., mechanical aeration) and environmental impacts, such as greenhouse gases emissions, nutrient losses, and secondary pollution from chemicals. Alternatively, biological treatment with microalgae can be an effective technology for removing nutrients and other contaminants with less consumption of energy, greenhouse emissions and costs; therefore, providing a more sustainable WWT. The algae (through photosynthesis) provide the O_2_ used by heterotrophic microorganisms to oxidize or assimilate organic carbon, nitrogen and phosphorus [8,9,10]. Poultry effluent’s characteristics are a suitable cultivation medium for mixotrophic microalgae, with carbon/nitrogen (C/N) and nitrogen/phosphorus (N/P) ratios favorable for microalgae growth [11].

The use of microalgae for WWT and removal of heavy metals has been extensively investigated in the past decade [12,13], and their accumulation of heavy metals has been used as biomonitors of metal pollution [14]. Bioremediation by microalgae has been extensively studied for several effluents, including municipal, agricultural and industrial ones [10,15,16,17,18,19,20,21,22]. Markou et al. studied the use of diluted poultry litter leachate with *Arthrospira platensis* and *Chlorella vulgaris* [23]. *C. vulgaris* had a better performance, being able to grow in 1/10 dilution reaching concentrations of 1.76–1.87 g/L and with a superior reutilization of nutrients. In a 29-day study with *Scenedesmus obliquus* (now known as *Tetradesmus obliquus*) grown in slaughterhouse poultry effluent, yields of 2.9 g/L and remediations of 100% for total nitrogen and phosphorus were achieved [18]. Furthermore, *T. obliquus* has been extensively used for WWT [15] because it is very robust and can grow in a wide range of pH, temperature and irradiance under heterotrophic and mixotrophic conditions [24].

The microalgae biomass obtained after WWT has been shown to be valuable bioactive compounds for the generation of bioenergy [15,16,20,25], animal feed [26,27], fertilizers [28,29], biostimulants [30,31,32,33,34,35], and biopolymers [36]. However, the use of WW to grow microalgae restricts their application in products intended for human consumption. However, if an extraction step combining the breakdown of the cell wall and the release of bioactive compounds with the elimination of contaminants, pathogens, and toxins, could allow this biomass to become a cheaper and safe source of bioactive components for products for human and animal use. In the case of *T. obliquus*, because it possesses a very robust cell wall, it requires harsher methods to break the cells [37].

Pressurized water or subcritical water (pressure usually between 10–60 bar) at temperatures in the range of 100 to 374.15 °C is used as a solvent for components of different ranges of polarity, from polar to non-polar. In a subcritical state, the dielectric constant of water can be reduced to the value of organic solvents [38]. Subcritical water (SW) extraction represents a superior extraction technique compared to conventional ones using organic solvents, because it is carried out in shorter times and without the generation of toxic waste. The selection of parameters enables the adjustment of selectivity of extracts’ characteristics including chemical composition and biological activity. At SW temperatures, chemical transformations can occur such as decomposition or formation of new components [39]. Some of the newly formed components can have a significant biological potential, while others can represent products of decomposition due to excessively high temperatures that could decrease the quality of the extracts. The general rule is to apply as high temperatures as possible to provide the maximal efficiency of extraction of components of interest, without the decomposition by-products [40,41,42]. In addition, the application of high temperatures has a destructive impact on microorganisms, including pathogens [43]. Therefore, SW can have a dual-purpose application extracting the bioactive components and reducing or eliminating pathogenic microorganisms. This potential was already shown in a previous study by Ferreira et al., where SW was applied for the extraction of *T. obliquus* biomass obtained from brewery WWT [16], drastically reducing the microbiological contamination of the initial biomass and producing microbiologically safe liquid extracts.

Therefore, the goal of this study was to establish a green sustainable procedure that integrates (1) the use of *Tetradesmus obliquus* in poultry WWT and (2) the obtained biomass processed by SW extraction with the aim of obtaining safe liquid extracts and solid residue towards zero waste. Furthermore, the presence of valuable bioactive compounds was monitored as well as the metal contents and microbiological profile of the biomass, extract, and solid residue, to access their potential and safeties for further applications.

## 2. Results and Discussion

### 2.1. Wastewater Treatment

*T. obliquus* grown in poultry WW achieved an average productivity of 37 ± 3 mg/(L·d) with a specific growth rate of 0.12 ± 0.00 d^−1^, resulting in high removal efficiencies of COD (84%), total Kjeldahl nitrogen (TKN—77%), and phosphate (50%) (Table 1). Nonetheless, these results are lower than the biomass productivities obtained by Oliveira et al. [18] and Ferreira et al. [15] (100 mg/(L·d) for 29 days) and Viegas et al. [32] (95 mg/(L·d) for 10 days). The same can be said for the removal efficiencies with values higher than 96 [15] and 70% [32] for all pollutants. Although the pollutant removal can be attributed to the nutrient assimilation by the microalga, some physico-chemical processes mediated by pH played an important role, such as ammonia volatilization and phosphate precipitation [44]. COD removal can be attributed to the assimilation of organic carbon through mixotrophic processes, which are common for *T. obliquus*, and the oxidation of organic matter by bacteria, using the oxygen supplied by the algae photosynthetic activity. Despite the high removal efficiencies, the final values achieved after microalga cultivation are still not in accordance to European legislation (Directive 91/271/EEC) [45]. In terms of metals, *T. obliquus* had a significant effect on the removal of all analyzed metals (49–89%). The results were compared with the maximum allowable values given by BAT-AEL (EU 2016/902), and they were within the values (25 μg Cr/L, 50 μg Cu/L, 50 μg Ni/L, 300 μg Zn/L) [46]. However, an optimization of cultivation parameters, mainly light intensity, is necessary to improve microalga productivity and, consequently, the pollutant removal to assure all legal levels are met. 

Results of GC/MS screening analysis of poultry WW before and after microalga-based treatment are presented in Table S1 of the Supplementary Material. Most identified compounds in poultry WW belong to the group of monocarboxylic organic acids (C_6_–C_23_) and aldehydes (C_8_–C_18_) followed by aliphatic saturated and unsaturated hydrocarbons and methylated hydrocarbons with the chain length of C_12_–C_23_. Identified compounds containing oxygen functional groups cover ketones, alcohols, esters, and ethers while aromatics include phenols, biphenyls, and polycyclic aromatic hydrocarbons (PAHs). Lanosterol, tetracyclic triterpenoid from which animal and fungal steroids are derived are also detected in poultry WW. Carboxylic acids (e.g., hexanoic acid, heptanoic acid, benzoic acid, octenoic acid, n-decanoic acid, dodecanoic acid, tetradecanoic acid, pentadecanoic acid, n-hexadecanoic acid, oleic acid, heptadecanoic acid, etc.), aldehydes (octanal, nonanal, decanal, 4-decadienal) and other organics containing oxygen are present in poultry WW. They are probably intermediates of decomposition of more complex organic substances that originate from food, metabolic products, animal excreta, etc. Previous research indicated that this type of WW contains oil and grease, nitrogen and phosphorus, high concentrations of suspended solids, and also might have good biodegradability [47,48]. In addition, Wiyarno and Widyastuti indicated that several compounds in the poultry slaughterhouse WW were originated from the anaerobic decomposition of high molecular weight compounds such as proteins including mercaptans, skatoles, indoles, inorganic acids, aldehydes, ketones, and organic compounds containing nitrogen or sulfuric atoms are associated with bad odors [47]. Some of these compounds were identified in poultry WW (Supplementary Material, Table S1).

Treated water, on the other hand, contains significantly less monocarboxylic acids (benzoic acid and benzoic acid, 3-methyl-), aldehydes (2-heptadecenal, E-15-heptadecenal) and esters (isopropyl myristate) compared to the poultry WW. Some microalgae can consume organic carbon for growth, thus producing beneficial metabolites, such as lipids and pigments [49]. Ethers, biphenyls and terpenoids were not identified in the treated water. The absence of some organics compounds in treated water which were initially identified in poultry WW might be attributed to microalgae activity that removed the organics through different mechanisms including biodegradation, consumption and biosorption [50]. Algae cell structural constituents can bind with pollutants. Various functional groups including amino, carboxyl, sulphates, phosphates, and imidazoles can act as specific sites for binding with and removal of pollutants [51,52]. For example, absence of organic acids and some aldehydes in the treated water can be a result of biosorption, where electrostatic interaction and ion exchange might occur on the surface of microalgal biomass [53]. Organic acids could be present in the WW as anions and can interact with positively charged algae residues at the protein. Additionally, organic acid and aldehydes could form hydrogen bonds with other functional groups of microalgae [52]. However, a slightly higher number of hydrocarbons compounds and ketones were detected. Phenols and PAHs were detected in both poultry WW and treated water. Zhang et al. (2019) indicated that the lipid fractions play an important role in the sorption of PAHs on algal biomass. The biosorption is the first step of the removal of the present organic pollutants after which the biodegradation step can occur [54]. The presence of chlorinated hydrocarbons and phthalates in the treated water might be attributed to the metabolic transformations of organic matter in the presence of microalgae. It is important to underline that due to the high concentration and diversity of the present organic pollutants in the WW, the biodegradation mechanism is complex.

### 2.2. Biomass Composition

#### 2.2.1. Content of Polyphenols and Antioxidant Activity of Biomass Extract

The total content of phenolics present in the liquid extract obtained by SW was 1.073 mg GAE/mL, while the total flavonoid content was approximately 10 times lower (0.111 mg CAT/mL) (Table 2). This phenolic yield is higher than the ones determined in SW extracts (at 160 °C) obtained previously from the same *T. obliquus* biomass grown in a synthetic (Bristol) medium (0.433 mg GAE/mL) [55], and in brewery WW (0.397 mg GAE/mL) [56]. However, it is comparable to the yields obtained in SW extracts (at 170 °C) using biomass grown in brewery WW with CO_2_ supplementation (1.059 mg GAE/mL) [56]. For flavonoids, the present results (0.111 mg CE/mL, Table 2) are slightly higher compared to 0.023 mg CAT/mL for Bristol [55] and 0.053–0.067 mg CAT/mL for brewery WW [56]. 

The antioxidant activity of extracts from poultry-grown *T. obliquus* was 7.18 µg/mL at 160 °C, Table 2), which is similar to the ones from brewery-grown *T. obliquus* (7.98 µg/mL at 170 °C) [56]. However, when grown in Bristol [55] or brewery WW with CO_2_ [56], in near optimal conditions, this microalga seemed to have stronger antioxidant activities (4.81 and 6.57 µg/mL, respectively). It is interesting to notice that the same microalga grown in different media and conditions will display different antioxidant properties, suggesting the possibility of tailoring the biomass for diverse applications. Nonetheless, the *T. obliquus* extracts exhibited greater antioxidant activity than extracts of the medicinal wild garlic plant (*Allium ursinum* L.) obtained by SW extraction under optimized conditions (179 °C, 10 min and 1.09% of acid modifier; 13 µg/mL) [57]. 

#### 2.2.2. Organic Profile of Whole Biomass, Extract, and Residue

**Figure 1:**
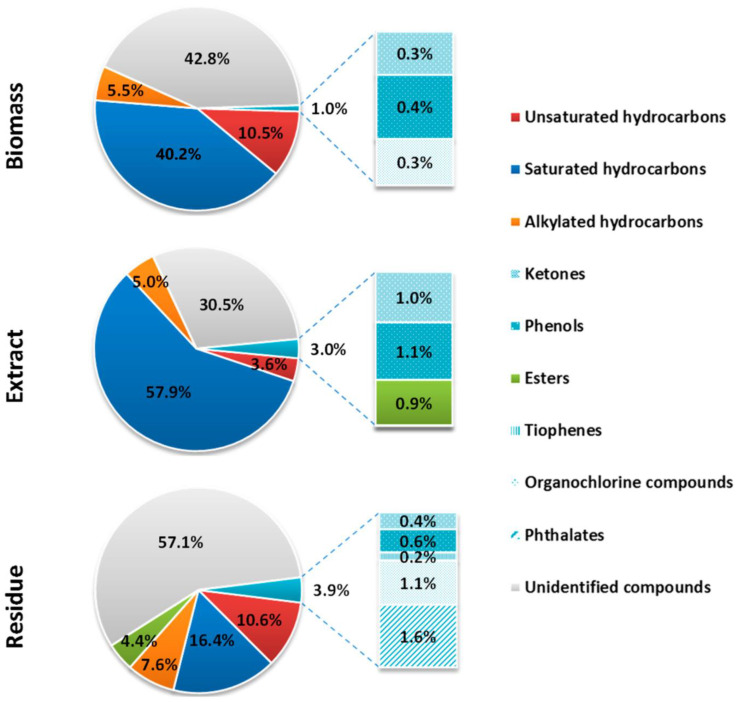
Organic compounds (% distribution based on the peak area) in the *Tetradesmus obliquus* biomass, extract, and solid residue, after subcritical water extraction (170 °C, 20 min, 30 bar).

The results of GC/MS screening analysis of biomass, and SW extract and residue are presented in Figure 1 (a more detailed list of compounds is given in Table S2 of Supplementary Material). *T. obliquus* biomass, SW extract, and residue were shown to be valuable sources of aliphatic (saturated and unsaturated) and alkylated (mostly methylated) hydrocarbons. In both biomass and extract, there was a predominance of hydrocarbons with the carbon chain lengths of C_12_–C_44_ (56–65%) where hexadecane was the most abundant compound (10–21%). The percentage of saturated hydrocarbons was 40.3, 57.9, and 16.4%, respectively, in biomass, extract, and residue. The percentage of unsaturated hydrocarbons was similar in both biomass and residue (10.5–10.6%), compared to 3.6% in the extract. The alkylated hydrocarbons represented 5 to 8% in all three samples. The presence of squalene was also determined in the biomass. This triterpenic compound possesses an exceptional biological importance as an antioxidant [58], an agent in neurodegenerative and cardiovascular diseases, and exhibits a beneficial impact on the function of liver, pancreas, and the immune system [59,60,61]. The use of squalene for cosmetic applications is limited because its primary source is shark liver oil. However, if microalgae can be a source of this compound, this would significantly contribute to the protection of this marine species [61,62,63,64].

Ketones and phenols were also determined in all three samples but were more abundant in the extract (around 1% each) than in the biomass and residue. Phenol, 2, 4-bis (1,1-dimethylethyl), which has a widespread application in several areas [65], was found in all three samples. This compound has proven antioxidant, antifungal [66], antibacterial [67], and citotoxic activity [68]. Due to its antifungal and antioxidant properties, it was demonstrated that it can be a potential food additive [66]. Furthermore, it was shown to have an allelopathic effect on germination and seedling growth of weedy plants under soilless conditions, as well as on the protection of fungal plant diseases (phytopathogenic fungi) [69,70]. 

SW can have the role of a catalyst for hydrolysis and other reactions, resulting in the formation of new components [71]. Esters were not present in the *T. obliquus* biomass but were in both the extract (0.9%) and residue (4.4%). Hexadecanoic acid ethyl and methyl esters were attributed with anti-inflammatory activity [72]. The latter also possesses antifibrotic [73] and antioxidant activity [74]. Moreover, Yu et al. indicated that methyl esters cause apoptosis and inhibit proliferation of human gastric cancer cell line, hence have the potential to be applied as anticancer agents in cancer treatment [75].

Phthalates and 3-Methyl-2-(3,7,11-trimethyldodecyl)thiophene were only detected in the residue (1.6 and 0.2%, respectively). Finally, organochlorine compounds were found in the biomass (0.3%) and the residue (1.1%), but not in the extract.

#### 2.2.3. Metal Content of Whole Biomass, Extract, and Residue

The contents of metals of microalga *T. obliquus* before and after SW extraction (extract and residue) are shown in Table 3. 

The metals present in the whole biomass are also present in the poultry WW, meaning that the microalga assimilated them during the treatment. The highest metal concentrations (2070 mg Fe/kg, 3530 mg Zn/kg, 91.9 mg Cu/kg and 88.3 mg Mn/kg) also correspond to the highest removal efficiencies (Table 1). Furthermore, no Co or As were present in the WW and, thus, they were also not present in the biomass. The SW extraction did not affect the metal content, meaning that most metals remained in the residue, except for Zn, which decreased by 78%.

A significant share of the organic material present in the extraction residue indicates the possibility of utilizing this material as animal feed or, after additional biological processing, soil conditioner or fertilizer in agriculture. Comparing the obtaining results with the Directive 2002/32/EC for feed, the residues of the extraction entirely meet the obligatory criteria [76]. For the application of this waste material in agriculture, the contents of heavy metals should be compared to the values prescribed by the Directive 86/278/EEC [77]. The obtained results of the content of heavy metals in the residues were under the prescribed values for the application in agriculture according to the EU directive.

#### 2.2.4. Microbiological Profile

To determine the impact of SW on microorganisms present in the biomass, the microbiological profiles of the biomass, extract, and residue were analyzed (Table 4).

The *T. obliquus* biomass had a confirmed presence of microorganisms with the total microbial count of 190 × 105 cfu/g. In addition, the presence of molds and yeasts as well as spores of anaerobic mesophilic bacteria was also determined. Considering the biomass was obtained in poultry WWT, the presence of indicators of fecal contamination, Enterobacteriaceae and members of the thermotolerant coliform group *Escherichia coli*, was also investigated and it amounted to <10 cfu/g. After extraction and exposure to 170 °C, the microorganisms in the extract and residue were eliminated, achieving the microbiological safety of both products. Using SW for the elimination of microorganisms was established in the previous study [16], in which brewery WWT biomass was extracted. The initial total microbial count was lower (64 × 103 cfu/g), which was expected considering the WW origin. Therefore, microbiologically safe clean extract and residue as a cheap resource of bioactive compounds were obtained using biomass from poultry in SW extraction. 

## 3. Materials and Methods

### 3.1. Poultry Wastewater Treatment

The wastewater used in the present work was obtained from a broiler chicken slaughterhouse from the company Avibom, S.A in Ramalhal (38°15′553.6″ N, −9°26′28.9″ W) located in the Torres Vedras, Portugal. This WW results from the cooking of the chicken remains not intended for human consumption (e.g., feathers, heads, feet, and viscera). The WW was characterized in terms of total Kjeldahl nitrogen (TKN), phosphate, and chemical oxygen demand (COD). TKN was determined according to Method 4500-Norg B [78]. Phosphate was determined using the ammonium molybdate spectrometric method (ISO 6878:2004) [79]. The COD determination was carried out by the potassium dichromate oxidation method—Method 5220-B [78]. 

### 3.2. Microalga, Cultivation Conditions

The microalga used was *Tetradesmus obliquus* (ACOI 204/07) from the ACOI Coimbra University Collection of Algae, Portugal. The microalga was cultivated in 4 L bubble-column photobioreactors in batch mode. The cultures were kept at room temperature (23–25 °C) and constant artificial illumination of 30 μmol/(m·s). Air was supplied at a flow rate of 0.1 vvm (L/(L·min)) for providing CO_2_ as carbon source and agitation. Microalga growth was monitored by daily measurements of optical density at 540 nm until cultures reached stationary phase (29 days). At the end, the biomass dry weight was determined using GF/C filters dried at 105 °C until constant weight. The microalga biomass was harvested by sedimentation followed by centrifugation (11,300× *g* for 10 min at 4 °C) and freeze-dried.

### 3.3. Extraction of Tetradesmus Obliquus Biomass

SW extraction was performed in a batch-type high-pressure extractor (Parr 4520, Parr Instrument Company, Moline, Illinois, USA) equipped with an anchor stirrer and an electric heater. The extraction conditions were 170 °C and 30 bar, for 10 min, with a biomass/water ratio of 1 g/10 mL. The extraction temperature was selected based on preliminary research [56]. The temperatures 120, 170, and 220 °C were tested and the extraction yield was measured. The highest extraction yield was determined at 170 °C. In addition, the extracts obtained at the highest temperature possessed a characteristic burnt smell. Pressure was provided using nitrogen. After the extraction, the extractor was immediately cooled to reach room temperature, and nitrogen was discharged from the extractor. Extraction was performed in triplicate. The liquid extracts and solid residue were separated by means of filtration and stored until the analysis.

### 3.4. Determination of Total Phenols and Total Flavonoids Content

Total phenols (TP) and total flavonoids (TF) content in obtained extract were measured according to the procedures described by Kähkönen et al. [80] and Markham et al. [81], respectively. Absorbance was measured at 750 nm (TP) and 510 nm (TF) (6300 Spectrophotometer, Jenway, Staffordshire, UK). Standard calibration curves were prepared using gallic acid and catechin as standard compounds and results are expressed as mg of gallic acid equivalents (GAE) per g of extract and mg of catechin equivalents (CAT) per g of extract, for TP and TF content, respectively. All the measurements were performed in triplicate.

### 3.5. Determination of Antioxidant Activity

The antioxidant activity of extract was analyzed using the 2,2-diphenyl-1-picrylhydrazyl (DPPH) assay [82]. Different extract volumes were mixed with 95% methanol and 90 μM DPPH solution. After the 60 min incubation at room temperature, absorption was measured at a wavelength of 515 nm. The antioxidant activity was expressed as IC_50_ value which represents the concentration of the extract which inhibits 50% DPPH radicals. All the measurements were performed in triplicate.

### 3.6. Gas Chromatography-Mass Spectrometry (GC-MS) Screening Analysis 

Qualitative screening of nonpolar and semipolar compounds in microalga biomass, SW residues, and SW liquid extracts were conducted using GC/MS analysis. Samples for GC/MS analysis were prepared according to the EPA 9071B method guidelines for n-hexane extractable material [83], commonly used for microalgae biomass analysis [84,85]. The method was modified with an additional step, where the samples were subjected to extraction using dichloromethane to cover a wider range of nonpolar to semipolar compounds. Samples were extracted by ultrasounds (EPA 3550b) [86]. Purification of samples was carried out using silica gel column, extracts were eluted and evaporated under nitrogen blowdown technique using TurboVap^®^ II Automated Solvent Evaporation System (Biotage, Uppsala, Sweden).

An Agilent Technologies 7890A gas chromatograph with a 5975C quadrupole mass spectrometer (Santa Clara, CA, USA) with HP-5MS column (30 m × 0.25 mm × 0.25 µm, J&W Scientific) was used for screening analysis. The chromatographic conditions were as follows: inlet temperature: 250 °C, splitless injection mode; initial oven temperature was 70 °C (2 min), followed by 25 °C/min to 150 °C; 3 °C/min to 200 °C, and 8 °C/min 280 °C (10 min). The temperature of the MS Source was 230 °C, and MS Quad was 150 °C. The MS was set at scan mode using a scan range from 50 to 450 m/z. Results of qualitative GC-MS screening analysis were evaluated using Agilent MSD Productivity ChemStation software and the NIST05 Mass Spectral Library. The study presents only compounds identified with a probability of a minimum of 80%. Additionally, a semi-quantitative analysis was performed based on the area% of each identified compound.

### 3.7. Determination of Metal Content

The chemical extraction for the determination of pseudo-total metal content in the solid phase and liquid extracts was performed according to the EPA method 3051A (2007) [87]. Moreover, 0.5 g of solid phase and 5 mL of liquid extracts were digested with the addition of the 10 mL HNO_3_ and HCl (3:1) in microwave unit (Milestone Microwave Extraction System, Start E). After digestion, the extract was filtered in the 25 mL flask. Metal content in the digested samples was then determined using the ICP-MS technique (Agilent Technologies 7700 Series ICP-MS, Santa Clara, CA, USA).

### 3.8. Microbiological Analysis

A 10 g portion of each sample was aseptically weighed and homogenized with 90 mL of buffered peptone water (HiMedia, Mumbai, India) using a Stomacher lab-blender (AES, Bruz, France). Serial decimal dilutions were prepared with the same diluent, and duplicate counting plates were prepared using appropriate dilutions. For pour plating, 1 mL aliquots of the dilutions were mixed with molten (47 °C) media and poured into plates. After incubation at appropriate temperatures, the colonies that appeared on the selected plates were counted as colony-forming units cfu/g or cfu/mL sample.

Microbiological determination of total aerobic microbial count, total yeast and mold count, *Enterobacteriaceae* and *Escherichia coli* was performed according to ISO standard microbiological methods (ISO 4833-1:2013 [88], ISO 21527-1:2008 [89], ISO 21527-2:2008 [90], ISO 21528-2:2004 [91] and ISO 16649-2:2001 [92]). Spores of anaerobic mesophilic bacteria were determined on nutrient agar (HiMedia) incubated under anaerobic condition at 30 °C for 48 h after 5 min in boiling water. Each test was performed in triplicate. 

## 4. Conclusions

This work aimed to couple poultry WWT using *T. obliquus* with the production of microbiologically safe products within a sustainable biorefinery process towards zero waste. Microalgae, particularly *T. obliquus*, have been shown to have an adequate biomass composition for bioenergy production and possess bioactive compounds with biofertilizer and biostimulant properties. However, the production of microalgae using wastewater as a source of water and nutrients could compromise the safety of the microalga biomass, mainly due to the presence of heavy metals and pathogens. The use of SW extraction allows the disruption and release of valuable compounds, such as antioxidants (phenols and flavonoids) and organic compounds with commercial interest, while simultaneously disinfecting the biomass and improving its quality to comply with legislation. 

The combination of using wastewater from different origins, the selection of robust microalgae species for effective WWT and adequate composition, and the green technologies acting at harsh conditions (eliminating pathogens and contaminants) to extract the valuable compounds could be the strategy for the future of microalgae biotechnology.

## Figures and Tables

**Table 1:** Removal efficiency by *Tetradesmus obliquus* in poultry wastewater (WW) in terms of chemical oxygen demand (COD), total Kjeldahl nitrogen (TKN), phosphate (PO_4_^3−^) and metals.

	Poultry WW	Treated Water	Removal (%)
**COD (mg O_2_/L)**	1950	268 ^1^	84
**TKN (mg N/L)**	277	63.8 ^1^	77
**PO_4_^3−^ (mg P/L)**	22.7	11.1 ^1^	50
**Metals (µg/L)**			
**Cr**	20.2	6.9	66
**Mn**	566	161	72
**Fe**	4040	511	87
**Co**	<1	<1	-
**Ni**	21.3	11.0	48
**Cu**	160	35	78
**Zn**	1140	128	89
**As**	<1	<1	-
**Cd**	<0.5	<0.5	-
**Pb**	7.83	3.98	49

^1^ Directive 91/271/EEC: COD—125 mg O_2_/L; TKN—15 mg N/L; PO_4_^3−^—2 mg P/L.

**Table 2:** Content of total phenols (TP), total flavonoids (TF) and antioxidant activity (expressed as IC_50_ value) of extract obtained by subcritical water of *Tetradesmus obliquus* biomass (170 °C, 20 min, 30 bar).

TP (mg GAE/mL)	TF (mg CE/mL)	IC_50_ (µg/mL)
1.073 ± 0.015	0.111 ± 0.002	7.18 ± 0.34

**Table 3:** Metal content present in *Tetradesmus obliquus* whole biomass, extract and residue following subcritical water extraction (170 °C, 20 min, 30 bar).

Metal	*T. obliquus*		
	Whole Biomass (mg/kg)	Extract (µg/L)	Residue (mg/kg)
Cr	16.2	145	16.14
Mn	88.3	369	90.74
Fe	2070	7890	1949.26
Co	<1	<1	<0.5
Ni	14.8	155	21.05
Cu	91.9	201	79.82
Zn	3530	587	760.12
As	<1	<1	<0.5
Cd	0.58	<0.5	<0.25
Pb	2.30	70.7	4.73

**Table 4:** Microbiological profile of *Tetradesmus obliquus* biomass, subcritical water (170 °C, 20 min, 30 bar) extract and residue. Results are expressed in colony-forming units (cfu) per g (biomass and residues) or mL (extracts).

*T. obliquus*	TAMC ^1^	Molds and Yeasts	Enterobacteriaceae	*E. coli*	SAMB ^2^
Whole biomass (cfu/g)	190 × 10^5^	300 (molds) <10 (yeasts)	<10	<10	12 × 10^2^
Extract (cfu/mL)	<1	<1	<1	<1	<1
Residue (cfu/g)	<10	<10	<10	<10	<10

^1^ TAMC—Total microbial count; ^2^ SAMB—Spores of anaerobic mesophilic bacteria.

## Data Availability

The authors declare that there are no associated data to this paper.

## Supplementary Materials

The following supporting information can be downloaded at: https://www.mdpi.com/article/10.3390/molecules28052397/s1, Table S1: Results of GC/MS screening of organic compounds in poultry wastewater and treated water after wastewater treatment with *Tetradesmus obliquus* microalga; Table S2: Results of GC/MS screening of organic compounds in *Tetradesmus obliquus* biomass, extract, and residue after subcritical water extraction.

## Sample Availability

Samples of the compounds are not available from the authors.
