# Supplementary material for: Application of Green Technology to Extract Clean and Safe Bioactive Compounds from Tetradesmus obliquus Biomass Grown in Poultry Wastewater
Source: Molecules. 2023 Mar 6;28(5):2397. doi: 10.3390/molecules28052397 (PMC10005368; doi:10.3390/molecules28052397)
Supplement: Supplementary file 1 [file molecules-28-02397-s001.zip › molecules-2150251-supplementary.pdf]

Supplementary Material

Table S1. Results of GC/MS screening of organic compounds in poultry wastewater and treated water after wastewater treatment with *Tetradesmus obliquus* microalga.

Identified compounds	Molecular formula	Poultry wastewater	Treated water
	HYDROCARBONS		
	Hydrocarbons with saturated bonds		
Undecane	$C_{11}H_{24}$	-	+
Dodecane	$C_{12}H_{26}$	+	+
Tridecane	$C_{13}H_{28}$	+	+
Tetradecane	$C_{14}H_{30}$	+	+
Pentadecane	$C_{15}H_{32}$	+	+
Hexadecane	$C_{16}H_{34}$	+	+
Heptadecane	$C_{17}H_{36}$	+	+
Octadecane	$C_{18}H_{38}$	-	+
Eicosane	$C_{20}H_{42}$	-	+
Tetracosane	$C_{24}H_{50}$	-	+
Octacosane	$C_{28}H_{58}$	-	+
Total No.		***6***	***11***
	Hydrocarbons with unsaturated bonds		
2-Tetradecene, (E)-	$C_{14}H_{28}$	+	+
1-Tetradecene	$C_{14}H_{28}$	-	+
1-Hexadecene	$C_{16}H_{32}$	+	+
Z-8-Hexadecene	$C_{16}H_{32}$	-	+
3-Eicosene, (E)-	$C_{20}H_{40}$	+	-
10-Heneicosene (c,t)	$C_{21}H_{42}$	+	-
9-Tricosene, (Z)-	$C_{23}H_{46}$	+	-
Total No.		***5***	***4***
	Alkylated hydrocarbons		
Tetradecane, 2-methyl-	$C_{15}H_{32}$	+	+
2-Methyl-Z-4-tetradecene	$C_{15}H_{30}$	+	-
Dodecane, 2,6,11-trimethyl-	$C_{15}H_{32}$	-	+
Pentadecane, 5-methyl-	$C_{16}H_{34}$	-	+
Tridecane, 6-propyl-	$C_{16}H_{34}$	-	+
Hexadecane, 2-methyl-	$C_{17}H_{36}$	-	+
Heptadecane, 2-methyl-	$C_{18}H_{38}$	-	+
Octadecane, 2-methyl-	$C_{19}H_{40}$	-	+
Nonadecane, 3-methyl-	$C_{20}H_{42}$	-	+
Total No.		***2***	***8***
	KETONES		

Identified compounds	Molecular formula	Poultry wastewater	Treated water
2-Undecanone	$C_{11}H_{22}O$	-	+
Benzophenone	$C_{13}H_{10}O$	-	+
2-Tridecanone	$C_{13}H_{26}O$	+	+
2-Tetradecanone	$C_{14}H_{28}O$	-	+
Total No.		***1***	***4***
**PHENOLS**			
Phenol, 4-methyl-	C_7H_8O	+	-
Phenol, 4,5-bis(1,1-dimethylethyl)	$C_{14}H_{22}O$	+	+
Phenol, 2,4-di-t-butyl-6-nitro-	$C_{14}H_{21}NO_3$	-	+
Total No.		***2***	***2***
**ORGANIC ACIDS**			
Pentanoic acid, 4-methyl-	$C_6H_{12}O_2$	+	-
Hexanoic acid	$C_6H_{12}O_2$	+	-
Benzoic acid	$C_7H_6O_2$	-	+
Hexanoic acid, 4-methyl-	$C_7H_{14}O_2$	+	-
Heptanoic acid	$C_7H_{14}O_2$	+	-
Benzoic acid, 3-methyl-	$C_8H_8O_2$	-	+
2-Octenoic acid	$C_8H_{14}O_2$	+	-
n-Decanoic acid	$C_{10}H_{20}O_2$	+	-
Dodecanoic acid	$C_{12}H_{24}O_2$	+	-
Tetradecanoic acid	$C_{14}H_{28}O_2$	+	-
Pentadecanoic acid	$C_{15}H_{30}O_2$	+	-
Z-7-Hexadecenoic acid	$C_{16}H_{30}O_2$	+	-
n-Hexadecanoic acid	$C_{16}H_{32}O_2$	+	-
Heptadecanoic acid	$C_{17}H_{34}O_2$	+	-
Oleic Acid	$C_{18}H_{34}O_2$	+	-
9,12-Octadecadienoic acid (Z,Z)-	$C_{18}H_{32}O_2$	+	-
6-Octadecenoic acid, (Z)-	$C_{18}H_{34}O_2$	+	-
Octadec-9-enoic acid	$C_{18}H_{34}O_2$	+	-
22-Tricosenoic acid	$C_{23}H_{44}O_2$	+	-
Total No.		***17***	***2***
**ESTERS**			
Hexanoic acid, hexyl ester	$C_{12}H_{24}O_2$	+	-
Isopropyl Myristate	$C_{17}H_{34}O_2$	-	+
(Z)-9-Octadecenoic acid butyl ester	$C_{22}H_{42}O_2$	+	-
Total No.		***2***	***1***
**ETERS**			
Methyl 4,6-decadienyl ether	$C_{11}H_{20}O$	+	-
Total No.		***1***	***0***
**ALDEHYDES**			
Octanal	$C_8H_{16}O$	+	-

Identified compounds	Molecular formula	Poultry wastewater	Treated water
Nonanal	$C_9H_{18}O$	+	-
Decanal	$C_{10}H_{20}O$	+	-
2,4-Decadienal, (E,E)-	$C_{10}H_{16}O$	+	-
2-Heptadecenal	$C_{17}H_{32}O$	-	+
E-15-Heptadecenal	$C_{17}H_{32}O$	-	+
9,17-Octadecadienal, (Z)-	$C_{18}H_{32}O$	+	-
Total No.		***5***	***2***
**ALCOHOLS**			
1-Dodecanol, 3,7,11-trimethyl-	$C_{15}H_{32}O$	-	+
Phytol	$C_{20}H_{40}O$	+	-
Total No.		***1***	***1***
**BIPHENYLS**			
2,2'-Dimethylbiphenyl	$C_{14}H_{14}$	+	-
Benzene, 1-methyl-4-(phenylmethyl)	$C_{14}H_{14}$	+	-
Total No.		***2***	***0***
**TERPENOIDS**			
Lanosterol	$C_{30}H_{50}O$	+	-
Total No.		***1***	***0***
**ORGANOCHLORINE COMPOUNDS**			
Octadecane, 1-chloro-	$C_{18}H_{37}Cl$	-	+
Nonadecane, 1-chloro-	$C_{19}H_{39}Cl$	-	+
Total No.		***0***	***2***
**POLYCICLIC AROMATIC HYDROCARBONS**			
Anthracene	$C_{14}H_{10}$	-	+
2,6-Diisopropylnaphthalene	$C_{16}H_{20}$	+	+
Total No.		***1***	***2***
**PHTHALATE**			
Dibutyl phthalate	$C_{16}H_{22}O_4$	-	+
1,2-Benzenedicarboxylic acid, mono(2-ethylhexyl) ester	$C_{16}H_{22}O_4$	-	+
Phthalic acid, bis(7-methyloctyl)ester	$C_{26}H_{42}O_4$	-	+
Phthalic acid, decyl nonyl ester	$C_{29}H_{48}O_4$	-	+
Total No.		***0***	***4***

Table S2. Results of GC/MS screening of organic compounds in *Tetradesmus obliquus* biomass, extract, and residue after subcritical water extraction.

Identified compounds	Molecular formula	Biomass	Extract	Residue
	**HYDROCARBONS**			
	Hydrocarbons with saturated bonds			
Dodecane	$C_{12}H_{26}$	-	+	+
Tridecane	$C_{13}H_{28}$	-	+	+
Tetradecane	$C_{14}H_{30}$	+	+	+
Pentadecane	$C_{15}H_{32}$	+	+	+
Hexadecane	$C_{16}H_{34}$	+	+	+
Heptadecane	$C_{17}H_{36}$	-	+	+
Octadecane	$C_{18}H_{38}$	+	+	+
Nonadecane	$C_{19}H_{40}$	-	+	+
Eicosane	$C_{20}H_{42}$	+	+	+
Heneicosane	$C_{21}H_{44}$	+	+	+
Docosane	$C_{22}H_{46}$	+	-	-
Tetracosane	$C_{24}H_{50}$	+	+	-
Heptacosane	$C_{27}H_{56}$	+	+	+
Octacosane	$C_{28}H_{58}$	+	+	+
Tetratriacontane	$C_{34}H_{70}$	-	-	+
Tetratetracontane	$C_{44}H_{90}$	+	-	+
Total No.		***11***	***13***	***14***
	Hydrocarbons with unsaturated bonds			
1-Dodecene	$C_{12}H_{24}$	-	+	-
1-Tetradecene	$C_{14}H_{28}$	-	+	+
1-Hexadecene	$C_{16}H_{32}$	+	+	+
3-Heptadecene, (Z)-	$C_{17}H_{34}$	+	-	+
8-Heptadecene	$C_{17}H_{34}$	-	-	+
1-Octadecene	$C_{18}H_{36}$	+	+	+
5-Octadecene, (E)-	$C_{18}H_{36}$	-	-	+
1-Nonadecene	$C_{19}H_{38}$	+	-	+
Z-5-Nonadecene	$C_{19}H_{38}$	-	-	+
1,19-Eicosadiene	$C_{20}H_{38}$	-	-	+
1-Docosene	$C_{22}H_{44}$	+	+	-
9-Tricosene, (Z)-	$C_{23}H_{46}$	-	-	+
2,6,10,14,18,22-Tetracosahexaene	$C_{24}H_{38}$	-	-	+
1-Hexacosene	$C_{26}H_{52}$	-	-	+
9-Hexacosene	$C_{26}H_{52}$	+	-	-
Squalene	$C_{30}H_{50}$	+	-	-
17-Pentatriacontene	$C_{35}H_{70}$	-	-	+
Total No.		***7***	***5***	***12***
	Alkylated hydrocarbons			

Identified compounds	Molecular formula	Biomass	Extract	Residue
2-Undecene, 5-methyl-	$C_{12}H_{26}$	-	+	-
Dodecane, 2-methyl-	$C_{13}H_{28}$	+	+	-
Undecane, 5,7-dimethyl-	$C_{13}H_{28}$	-	+	-
Tetradecane, 2-methyl-	$C_{15}H_{32}$	+	+	+
Pentadecane, 3-methyl-	$C_{16}H_{34}$	+	+	+
Hexadecane, 2-methyl-	$C_{17}H_{36}$	+	+	+
Heptadecane, 3-methyl-	$C_{18}H_{38}$	+	+	+
Octadecane, 2-methyl-	$C_{19}H_{40}$	+	+	-
Nonadecane, 3-methyl-	$C_{19}H_{38}$	+	+	-
10-Methylnonadecane	$C_{20}H_{42}$	+	+	-
2-Hexadecene, 3,7,11,15-tetramethyl-, [R-[R*,R*-(E)]]-	$C_{20}H_{40}$	-	-	+
Heneicosane, 11-(1-ethylpropyl)-	$C_{26}H_{54}$	-	+	-
Total No.		***8***	***11***	***5***
		**KETONES**		
Benzophenone	$C_{13}H_{10}O$	+	+	+
2-Tridecanone	$C_{13}H_{26}O$	-	+	-
2-Tetradecanone	$C_{14}H_{28}O$	-	-	+
2-Nonacosanone	$C_{29}H_{58}O$	-	-	+
Total No.		***1***	***2***	***3***
		**PHENOLS**		
Phenol, 2,4-bis(1,1-dimethylethyl)	$C_{14}H_{22}O$	+	+	+
Total No.		***1***	***1***	***1***
		**ESTERS**		
Hexadecanoic acid, methyl ester	$C_{17}H_{34}O_2$	-	-	+
Hexadecanoic acid, ethyl ester	$C_{18}H_{36}O_2$	-	+	-
8-Octadecenoic acid, methyl ester	$C_{19}H_{36}O_2$	-	-	+
1,2-Benzenedicarboxylic acid, diisooctyl ester	$C_{24}H_{38}O_4$	-	+	+
Total No.		***0***	***2***	***3***
		**PHTHALATES**		
Dibutyl phthalate	$C_{16}H_{22}O_4$	-	-	+
Phthalic acid, butyl undecyl ester	$C_{23}H_{36}O_4$	-	-	+
Total No.		***0***	***0***	***2***
		**TIOPHENES**		
3-Methyl-2-(3,7,11-trimethyldodecyl)thiophene	$C_{20}H_{36}S$	-	-	+
Total No.		***0***	***0***	***1***
		**ORGANOCHLORINE COMPOUNDS**		
Dichloroacetic acid, heptadecyl ester	$C_{19}H_{36}Cl_2O_2$	-	-	+
2- Chloropropionic acid, octadecyl ester	$C_{21}H_{41}ClO_2$	+	-	+

Identified compounds	Molecular formula	Biomass	Extract	Residue
Heptacosane, 1-chloro-	$C_{27}H_{55}Cl$	-	-	+
Total No.		***1***	***0***	***3***
